# APC I1307K and clinical management: insights from UK Biobank association analysis of colorectal and other cancer risks in Ashkenazi and non-Ashkenazi whites

**DOI:** 10.1136/jmg-2025-110911

**Published:** 2025-08-27

**Authors:** Sophie Allen, Charlie F Rowlands, Andrew Latchford, Clare Turnbull, Laura Valle

**Affiliations:** 1 Division of Genetics and Epidemiology, The Institute of Cancer Research, London, England, UK; 2 The Polyposis Registry, St Mark’s Centre for Familial Intestinal Cancer, Saint Mark’s Hospital, London, England, UK; 3 Department of Surgery and Cancer, Imperial College London, London, England, UK; 4 Royal Marsden Hospital NHS Trust, London, England, UK; 5 Hereditary Cancer Programme, Catalan Institute of Oncology; Oncobell Programme, IDIBELL, Hospitalet de Llobregat, Barcelona, Spain; 6 CIBERONC, Madrid, Spain

**Keywords:** Genetic Testing, Germ-Line Mutation, Genetics, Population, Genetic Predisposition to Disease, Digestive System Neoplasms

## Abstract

**Background:**

*APC* c.3920T>A; p.Ile1307Lys (I1307K), prevalent in individuals of Ashkenazi Jewish (AJ) origin, has been associated with a modestly increased colorectal cancer (CRC) risk. Clinical recommendations for I1307K heterozygotes vary across countries and expert groups, reflecting differences in population frequencies, modest risk estimates and limited data in non-AJ individuals.

**Methods:**

We analysed UK Biobank data comprising 466 315 individuals (8944 with CRC), using genomic analysis to classify AJ and non-AJ ancestries.

**Results:**

I1307K was identified in 7.1% of AJ and 0.08% of non-AJ white participants. No significant association with CRC was observed in AJ (OR: 0.71; 95% CI: 0.17 to 2.95) or non-AJ white individuals (OR: 1.05; 95% CI: 0.50 to 2.22). The previously established OR of 1.7–1.8 for AJ individuals lies within our 95% CI, indicating underpowered results due to limited CRC cases. No significant associations were detected for other cancers. Unbiased, adequately powered CRC case–control studies in non-AJ populations would require cohorts far larger than current resources for feasible analysis.

**Conclusion:**

Clinical actionability of I1307K should prioritise risk stratification based on overall CRC risk and ancestry-dependent variant detection rates. However, management strategies need not differ by ancestry once a carrier is identified, as the biological impact of I1307K should be consistent across populations.

**WHAT IS ALREADY KNOWN ON THIS TOPIC**

- *APC* I1307K modestly increases colorectal cancer risk in Ashkenazi Jewish (AJ) individuals. Its relevance in non-AJ populations remains unclear, contributing to inconsistent clinical guidelines.

**WHAT THIS STUDY ADDS**

- This is the first prospective cohort-based study to assess cancer risk associated with *APC* I1307K using UK Biobank data. Using genomic information to assign ancestry, it found no statistically significant association between I1307K and CRC or other cancers in either AJ or non-AJ white individuals. However, previously reported CRC risk estimates for AJ carriers remain within the observed confidence intervals. The study also highlights the statistical limitations of assessing risk in non-AJ populations due to the variant’s rarity.

**HOW THIS STUDY MIGHT AFFECT RESEARCH, PRACTICE OR POLICY**

- These findings support ancestry-informed testing for variant detection but advocate for consistent clinical management across ancestries, given the likely shared biological effect. The study underscores the logistical challenges of conducting adequately powered studies for rare, moderate-risk variants.

## Introduction

The *APC* (NM_000038.6) c.3920T>A p.Ile1307Lys (I1307K) missense variant has been associated with a moderate/low increased risk of colorectal cancer (CRC) in individuals of Ashkenazi Jewish (AJ) descent, in whom the allele frequency is the highest (minor allele frequency (MAF): 3.7%). The level of CRC risk associated with this variant in the AJ population (OR: 1.7–1.8) is similar to the observed epidemiological risk of CRC for an individual with one first-degree relative affected with CRC. There is no evidence for increased risk of polyposis, and the age at cancer diagnosis in I1307K AJ heterozygotes is similar to the age at diagnosis in the general population (mean age: 60–70 years).^1^

Although *APC* I1307K is predicted benign (AlphaMissense: 0.141; REVEL: 0.233; CADD: 8.522), it is located in the β-catenin binding domain of APC and affects an (A)3(T)(A)4 sequence, converting it into an extended tract of eight adenosine nucleotides (A8). In vitro and in vivo studies performed in the late 1990s suggest that this modification causes the slippage of the polymerase during DNA replication, conferring an increased propensity for somatic truncating mutations in that allele.^2 3^ Likewise, a single in vitro study showed that *APC* I1307K modestly increases β-catenin-regulated transcription; that is, results in the moderate activation of Wnt signalling.^4^

In 2023, the International Society for Gastrointestinal Hereditary Tumours (InSiGHT) issued an evidence-based position statement on *APC* I1307K and cancer risk based on meta-analyses of available data,^1^ which included consensus recommendations for the clinical management of carriers of I1307K and identification of research gaps that needed to be addressed. The group recommended that clinical CRC surveillance be restricted to carriers of *APC* I1307K of AJ origin on the basis that the only evidence for association with CRC was in the AJ population. The InSiGHT group highlighted the need for well-designed, geographically matched case–control studies to be able to make a clinically informative statement regarding association of *APC* I1307K for non-AJ individuals. The scarcity of data to assess the risk of extracolonic cancers and the lack of robust methods used to define ancestry in the published studies were also highlighted as research priorities.

Here we present an analysis of the UK Biobank (UKB) prospective cohort study comprising 466 315 individuals, and a genomic principal component (PC) analysis to identify an AJ ancestry group,^5^ aimed to investigate the association of *APC* I1307K with CRC and other cancer types in AJ and non-AJ individuals. Previous studies that evaluate the risk of cancer in I1307K carriers had relied on self-reporting of AJ ancestry, and/or the country of birth of parents and grandparents when Jewish descent was suspected. Here, we used a PC analysis based on genomic data, which enabled systematic assigning of ancestry equivalently to study participants, both AJ and non-AJ and both CRC cases and those unaffected by CRC.

## Methods

### UKB individuals included in the analysis

Full autosomal sequence data were retrieved from all participants within the UKB.^6^ Individuals between 40 and 69 years of age were recruited to UKB, with lifelong linkage to the cancer registry, data from which are updated annually. Related individuals were excluded based on a kinship relatedness cut-off of 0.0884 (second degree relatives or closer) using PLINK2.^5 7^ A total of 466 315 individuals were retained after filtering.

### Ancestry assessment using PC analysis

Data from UKB individuals was projected onto the 25-PC space of a reference dataset of 202 Jewish individuals and 1572 non-Jewish individuals using the bigsnpr package.^5 8^ Following the methodology described in Prive *et al*, the robust geometric mean of the AJ population in the reference dataset was calculated, and the average distance of each Jewish population to this geometric mean generated.^5^ A distance threshold of 15 was deemed sufficient to assign an individual to the AJ group based on inspection of a distance histogram and to maximise the number of AJ reference individuals while limiting the number of non-AJ Jewish individuals. This distance threshold is different to the previously reported threshold of 12.5 and is due to recalibration using a different UKB dataset. Due to overlap in distance scores, the threshold encompassed 23/29 ‘Ashkenazi Jewish’, 2/10 ‘Italian Jewish’, 1/6 ‘Tunisian Jewish’ and 1/22 ‘Sephardi Jewish’ based on self-reported ethnicity for individuals in the reference dataset. Decreasing the threshold to include only self-reported AJ individuals (threshold=9.2) would only retain 4/29; likewise, increasing the threshold to include more AJ references would increasingly include other ethnic groups. A UKB individual was thus classified as ‘AJ’ if the average distance across PCs was equal to or below the distance threshold of 15, and ‘non-AJ’ if above the threshold.

Non-AJ ethnicities were defined using UKB data-field 21000, collapsed using top-level ethnicity (‘Asian’, ‘Black’, ‘Chinese’, ‘Mixed’ and ‘White’). Where ethnicity for an individual indicated multiple of the top-level ethnicities or an ethnicity of ‘Other’, an ethnicity of ‘NA’ was instead assigned. Sex was defined using UKB data-field 31. Age for stratified analysis was defined using the age at first diagnosis of the relevant cancer from the cancer registry (data-field 40008) or self-reported age (data-field 20007) for cases, and age at date of censor for this analysis, 30 November 2022, for controls. To protect the confidentiality of UKB participants in the stratified analyses, where possible, values presented from data reported in the UKB have been censored at a minimum of five individuals.

### *APC* I1307K genotyping and cancer data in UKB

Individuals were considered to be carriers of NM_000038.5: *APC* c.3920T>A; p.(Ile1307Lys), if a variant was extracted from the UKB population-level pVCF-formatted variant^9^ at the GRCh38 chr5 112 839 514 position with a T>A change, without overlap with deletions or insertions covering this region. Homozygous and heterozygous carriers of this variant were annotated against participant ID and filtered based on AJ versus non-AJ status.

Each phenotype was defined using a combination of the reported ICD9 and ICD10 codes (UKB data-fields 40 013 and 40006) and self-reported diagnoses (field 20001). The selected terms searched to assign each cancer phenotype are described in online supplemental table 1.

### Statistical analysis

Fisher’s exact test of independence and quantification of effect size using an OR were used to test and measure the difference in carrier frequency between phenotype-positive and phenotype-negative individuals for AJ and non-AJ carriers. The Bonferroni correction was used to identify the threshold of significance for the Fisher’s exact test. The OR and 95% CIs (lower 95% CI=LCI, upper 95% CI=UCI) were calculated as follows:


$$OR=\frac{a}{b}*\frac{d}{c}$$

$$\begin{array}{l} \text{LCI}=e^{\log(OR)-\left(1.96*\sqrt{\left(\frac{1}{a}+\frac{1}{b}+\frac{1}{c}+\frac{1}{d}\right)}\right)};\\ \;\text{UCI}=e^{\log(OR)+\left(1.96*\sqrt{\left(\frac{1}{a}+\frac{1}{b}+\frac{1}{c}+\frac{1}{d}\right)}\right)} \end{array}$$

where *a*=number of variant carriers with the phenotype of interest; *b*=number of variant carriers without the phenotype of interest; *c*=number of individuals without the variant but with the phenotype of interest; *d*=number of individuals without the variant and without the phenotype of interest.

## Results

Using PC analysis, we identified 2726 individuals as being of AJ ancestry. The frequency of the *APC* I1307K in the UKB AJs was 7.1% (193/2726), a similar frequency as reported in control AJ populations of published studies.^1^ 436 938 non-AJ white individuals, as assessed by PC ancestry analysis, were identified (online supplemental figure 1). The I1307K frequency in these non-AJ white individuals was 0.077% (336/436 938), a frequency comparable to that reported in gnomAD v.4.1.0 for non-Finnish Europeans (480/589 993; 0.081%). No homozygotes were identified in the non-AJ white populations of either UKB or gnomAD.

We identified 8944 CRC cases in UKB based on cancer registration and patient self-report, including both previous and prospectively ascertained diagnoses. Within the AJ population, the frequency of *APC* I1307K in those with a CRC diagnosis was 2/39 (5.1%) vs 191/2687 (7.1%) in AJ individuals without CRC (OR: 0.71, 95% CI: 0.17 to 2.95).

When considering non-AJ white individuals, no association of I1307K with CRC risk was identified: 7/8688 (0.08%) patients with CRC versus 329/428 250 (0.08%) individuals without CRC (OR: 1.05, 95% CI: 0.50 to 2.22) (table 1). There was no evidence for association in non-AJ non-white populations of I1307K with CRC or other cancers, either individually or collectively (online supplemental table 2).

**Table 1:** APC I1307K frequency in CRC, breast, prostate, pancreatic and all patients with cancer and patients without these phenotypes in the UK Biobank, and associated ORs for AJ and non-AJ white individuals (Bonferroni-adjusted threshold of significance=5.00×10^−3^)

Ancestry	Patients with cancer (I1307K/total)	Individuals w/o the corresponding cancer type (I1307K/total)	OR (95% CI)	Fisher’s exact p value
	**CRC**			
AJ	2/39 (5.1%)	191/2687 (7.1%)	0.71 (0.17–2.95)	1
Non-AJ white	7/8688 (0.08%)	329/428250 (0.08%)	1.05 (0.50–2.22)	0.84
	**Breast cancer**			
AJ	11/131 (8.4%)	182/2595 (7.0%)	1.22 (0.64–2.29)	0.49
Non-AJ white	14/18683 (0.07%)	322/418255 (0.08%)	0.97 (0.57–1.66)	1
	**Prostate cancer**			
AJ	7/85 (8.2%)	186/2641 (7.0%)	1.18 (0.54–2.60)	0.67
Non-AJ white	7/14159 (0.05%)	329/422779 (0.08%)	0.64 (0.30–1.34)	0.28
	**Pancreatic cancer**			
AJ	<5/9	192/2717 (7.1%)	1.64 (0.20–13.21)	0.48
Non-AJ white	<5/1460	336/435478 (0.08%)	NA	NA
	**All cancers**			
AJ	45/538 (8.4%)	148/2188 (6.76%)	1.26 (0.89–1.78)	0.19
Non-AJ white	62/87689 (0.07%)	274/349249 (0.08%)	0.90 (0.68–1.19)	0.50

AJ, Ashkenazi Jewish; CRC, colorectal cancer.

Additionally, there was no evidence for association in either AJ or non-AJ white individuals, when stratifying by age (online supplemental table 3) or by age band (online supplemental table 4).

## Discussion

This is the first time a population-based prospective cohort study has been used to assess the cancer risks associated with *APC* I1307K. No association with CRC, breast cancer, prostate cancer or pancreatic cancer was identified for either AJ or non-AJ white individuals (table 1).

The estimate of association of I1307K with CRC in AJ individuals is OR: 0.71 (95% CI: 0.17 to 2.95), meaning that the previously established estimate of association of OR: 1.7–1.8 lies within the 95% CI.^1^ The breadth of the CI reflects the comparatively modest numbers of AJ individuals in UKB, particularly the small number of AJ CRC cases, meaning this study is underpowered for analysis of association of CRC in AJ patients compared with the multiple previous case–control studies in AJ individuals. Therefore, this analysis does not inform or alter current understanding of the association of CRC with I1307K in AJ individuals.

The association of I1307K and CRC in non-AJ white individuals has long been a subject of debate, as reflected by the InSiGHT recommendations. In a large case–control study by Forkosh *et al*,^10^ the frequency of the *APC* I1307K variant in non-AJ white patients with CRC was 64/26563 (0.24%): these were ascertained from clinical genetic testing data from Invitae, a US commercial laboratory, and described as being of self-reported French Canadian or white ethnicity (although the breakdown therein was not delineated). On comparison to non-AJ non-Finnish European controls from gnomAD v.2.1, in whom the frequency of I1307K was 73/58,918 (0.12%), the OR for I1307K and CRC was estimated to be 1.95 (95% CI: 1.39 to 2.73). The constitution of the gnomAD v.2.1 Non-Finnish European control series is described as: 40% NorthWestern European, 20% Swedish, 9% Southern European, 6% Bulgarian/Estonian and 26% other Non-Finnish European (based on genomically inferred ancestry). Thus, it is unclear the extent to which population structure (heterogeneity in ancestry between cases and controls) may have influenced the reported association, in particular given the recognised wide variation in frequency for the I1307K variant. Interestingly, this study also reports positive case–control association for the I1307K variant in non-AJ white individuals for melanoma (OR=2.54 (1.57–3.98)), female breast cancer (OR=1.73 (1.18–2.65)) and prostate cancer (OR=2.42 (1.45–3.94)). Notably, in the AJ partition of the analysis (using Invitae data for AJ cases of self-reported ancestry and gnomAD v.2.1 for AJ controls), the association with CRC was significant (145/1306 (11.1%) in cases and 342/4918 (6.9%) in controls, OR=1.67 (1.36–2.05)), while there was no significant association in any of these other cancers in AJ individuals despite well-powered analyses.

We have presented data from UKB on I1307K in 8688 non-AJ white CRC cases and 428 250 non-AJ white controls with OR for association between I1307K and CRC being 1.05 (95% CI: 0.50 to 2.22). The only other study, to our knowledge, of *APC* I1307K in (non-AJ) CRC cases and controls from the same geographical area is a case–control study from Spain comprising 4072 patients with CRC and 2739 unaffected controls. This also found no evidence of association with *APC* I1307K detected in 7/4072 (0.17%) patients with CRC versus 4/2739 (0.15%) controls (OR: 1.18; 95% CI: 0.3 to 4.0).^1^ It should be noted that no ancestry assessment was performed in this study. Compared with this analysis, our study is more than sevenfold larger overall, having twice the number of cases and 150-fold the number of controls, as well as being a cohort with systematic ascertainment and genotyping of participants agnostic to disease status and ancestry. Nevertheless, the rarity of I1307K in non-AJ white individuals means our analyses still categorically lack power to demonstrate or confidently refute association of *APC* I1307K with CRC in non-AJ populations. Lack of power was in part driven by the frequency of I1307K in non-AJ white controls in UKB of 0.08% being lower than that reported in the other series (noting technical parameters for calling from whole-exome sequencing (WES) of I1307K in UKB were of high quality and the frequency of I1307K in the AJ population of 7.1% (193/2726) is wholly consistent with other AJ frequency estimates). Compared with the Spanish series described by Valle *et al* in which the frequency of I1307K in the control population was 0.15%, the lower frequency observed in non-AJ whites from UKB may be a reflection of the application of a PC-based selection of non-AJ whites, and/or due to a remaining—mostly Sephardi—Jewish genetic pool in Spain. Considering that the AJ population in gnomAD v.2.1 was likewise delineated via PC analysis, the 50% higher frequency of I1307K (0.12%) in the non-AJ non-Finnish Europeans group thus likely reflects heterogeneity in I1307K frequency across non-AJ white populations. This geographic heterogeneity should be considered when reporting I1307K case–control association studies, as previously highlighted by the InSiGHT group.

This highlights a consideration regarding the threshold distance used to define AJ ancestry. We had recalibrated the genetic distance using the full UKB dataset in order to maximise the number of AJ reference individuals and minimise non-AJ reference individuals included within this genetic distance. Of note, as our genetic distance is modestly higher than previously used,^5^ there is a hypothetical possibility of inclusion as AJ in our UKB analysis of non-Ashkenazi heritage Jewish individuals or those of more dilute AJ heritage. Different methods in defining AJ heritage, with varying genetic approaches versus self-report, add potential additional complexity to interpretation of the literature.

As mentioned in the Introduction section, the mechanism of tumourigenesis ascribed to *APC* I1307K lies in the A8 tract, which is prone to mispairing and slippage during DNA replication, thereby creating frameshift changes in that region. The only somatic variant observed in the A8 tract is c.3924_3925insA, which translates into a frameshift change. *APC*, as a classic tumour suppressor, requires two hits to inactivate its tumour suppressor activity, and based on the location of the somatic c.3924_3925insA, loss of the heterozygosity (LOH) of the wildtype allele is expected. Thus, sequencing of colorectal tumour tissue for c.3924_3925insA to identify LOH of the other allele in non-AJ white I1307K variant carriers would also be a potential approach by which to explore aetiologic implication. However, in AJ heterozygotes, only ~1/3 of the CRCs harbour mutations in the A8 tract,^2 3 11 12^ and cooccurrence of the two somatic *APC* hits (c.3924_3925insA and LOH of the wildtype allele) is identified in approximately 10% of the CRCs,^3 11^ suggesting that many of these tumours are not related to the germline *APC* I1307K variant. This observation is entirely consistent with the modest OR for association with CRC of germline I1307K: where the magnitude of association and accordant attributable fraction are modest, absence of the classical aetiological molecular hallmarks in a significant proportion of tumours in individuals with the germline aberration is well-described in the context of different molecular phenomena (such as LOH and mutational signatures) in tumour types of low ‘aetiologic index’.^13^ Nevertheless, along with population cohorts, such as UKB, typically collecting constitutional DNA only, this factor would render investigation of tumours in non-AJ white I1307K variant carriers even more logistically implausible.

While analysis of a prospectively ascertained cohort obviates the potential for inflation from population structure (ancestry bias between cases and controls), the low frequency of both I1307K and of CRC in any broad prospective cohort study renders prohibitive the size of dataset required to provide a definitive answer to this question. UKB is noteworthy for having restricted recruitment to individuals aged 40–69 in order to enhance rates of accrual of cases of late-onset diseases; this cohort has now attained 14–18 years of maturation and the frequency of CRC is only 2%. For cohorts of young age of recruitment, for example All of Us, accrual of sufficient cases of CRC will require decades of follow-up even despite a larger overall cohort size. Assuming equivalent maturity, accrual and age structure to UKB (ie, a frequency of CRC of 2%), a cohort three times the size of UKB (1 495 550 individuals) would be required to provide 80% power for demonstration at 95% significance of an OR of 1.7 (assuming a baseline frequency of I1307K of 0.08%).

Early studies performed in AJ and non-AJ Jews identified a common haplotype for carriers with I1307K, regardless of their ethnic origin, suggesting a common origin.^14 15^ In line with this, Niell *et al* identified a common progenitor haplotype in I1307K individuals of Ashkenazi, Sephardi and Arab descent ascertained in Israel.^16^ It is highly plausible that the founder haplotype expanded to other communities that came in contact with AJs or other Jewish populations that carried I1307K, although to our knowledge, no haplotype analysis in non-Jewish individuals living outside Israel has been reported.

Clinical guidance regarding *APC* I1307K is currently rather contradictory. The guidelines of the National Comprehensive Cancer Network (NCCN v.3.2024) recommend ‘high-quality colonoscopy screening’ every 5 years (beginning at age 40, or 10 years before the youngest age at CRC diagnosis in a first-degree relative when there is family history of CRC), for all *APC* I1307K carriers regardless of ancestry. By contrast, the UK Cancer Genetics Group (UKCGG), a constituent group of the British Society of Genomic Medicine, recommended that the I1307K variant is not reported as part of the UK National Health Service (NHS)-funded diagnostic *APC* testing, regardless of the individual’s ancestry (based on evaluation in the context of health priorities, opportunity cost and equity of access within the UK NHS). The rationale for this position argued on the basis of the modest magnitude of CRC risk conferred by *APC* I1307K.^17^ By contrast, InSiGHT recommends that enhanced screening for CRC in *APC* I1307K heterozygotes/homozygotes should be restricted to individuals of AJ ancestry (as per enhanced screening offered to individuals with a first-degree relative affected with CRC), but that there should be no enhanced screening for non-AJ I1307K individuals because the risk association has not been proven for non-AJ ethnicities.

Overall, it would appear most likely that there is a single I1307K haplotype that confers modest susceptibility to CRC (and possibly other cancers), but its frequency outside the AJ population is too low to demonstrate statistically significant association with cancers within a single cohort. Our data demonstrate that adequately powered, unbiased case–control analysis of I1307K to assess association with CRC in the non-AJ white population would require a cohort of a size and maturity not plausibly feasible for decades. It is thus rational that (1) actionability of I1307K (ie, incorporation for CRC risk stratification and concomitant alteration in clinical management) should be determined on the basis of the (modest) associated risk; (2) if I1307K is deemed clinically actionable, strategies for testing I1307K may be informed by ancestry (on account of differential detection rates). It is not, however, immediately rational that the management in those with I1307K should be informed by their ancestry.

## Supplementary material

10.1136/jmg-2025-110911 online supplemental file 1

## Data Availability

Data may be obtained from a third party and are not publicly available. All data relevant to the study are included in the article or uploaded as supplementary information.
