# Supplementary material for: APC I1307K and clinical management: insights from UK Biobank association analysis of colorectal and other cancer risks in Ashkenazi and non-Ashkenazi whites
Source: J Med Genet. 2025 Aug 27;62(12):e110911. doi: 10.1136/jmg-2025-110911 (PMC12703324; doi:10.1136/jmg-2025-110911)

SUPPLEMENTARY MATERIAL

Supplementary Table 1: ICD9, ICD10 codes and self-reported diagnosis terms used to identify individuals diagnosed with each cancer phenotype.

	ICD9 Codes	ICD10 Codes	Self-reported search term
Colorectal Cancer	153.0, 153.1, 153.2, 153.3, 153.4, 153.5, 153.6, 153.7, 153.8, 153.9, 154.0, 154.1	C18.0, C18.1, C18.2, C18.3, C18.4, C18.5, C18.6, C18.7, C18.8, C18.9, C19, C20	“colo”, “rect”, “sigmoid”, “bowel”
Breast Cancer	174.0, 174.1, 174.2, 174.3, 174.4, 174.5, 174.6, 174.8, 174.9, 175.0, 175.9	C50.0, C50.1, C50.2, C50.3, C50.4, C50.5, C50.6, C50.8, C50.9	“breast”
Prostate Cancer	185	C61	“prostate”
Pancreatic Cancer	157.0, 157.1, 157.2, 157.3, 157.4, 157.8, 157.9	C25.0, C25.1, C25.2, C25.3, C25.4, C25.7, C25.8, C25.9	“pancrea”
All Cancers	140-165, 170-176, 179-209, 230-239	C00-C97, except C44 (non-melanoma skin cancer)	All indications except “non-melanoma skin cancer”

Supplementary Table 2: *APC* I1307K frequency in breast, prostate, pancreatic, and all cancer patients and patients without these phenotypes in the UK Biobank. Associated ORs for AJ and non-AJ individuals, stratified by ancestry, are provided. P-values were obtained using a Fisher's exact test (Bonferroni-adjusted threshold of significance: $1.67x10^{-3}$).

Ancestry	Cancer patients (I1307K/total)	Individuals w/o the corresponding cancer type (I1307K/total)	OR (95% CI)	Fisher's exact p-value
	Colorectal cancer			
AJ	<5/39	191/2687 (7.1%)	0.71 (0.17 — 2.95)	1
Non-AJ White	7/8688 (0.08%)	329/428250 (0.08%)	1.05 (0.50 – 2.22)	0.84
Non-AJ Black	<5/85	<5/7158	*NA*	*NA*
Non-AJ Asian	<5/73	13/8980 (0.1%)	*NA*	*NA*
Non-AJ Chinese	<5/15	<5/1432	*NA*	*NA*
Non-AJ Mixed	<5/44	9/2653 (0.3%)	*NA*	*NA*
	All cancers			
AJ	45/538 (8.4%)	148/2188 (6.76%)	1.26 (0.89 – 1.78)	0.19
Non-AJ White	62/87689 (0.07%)	274/349249 (0.08%)	0.90 (0.68 – 1.19)	0.50
Non-AJ Black	<5/894	<5/6349	*NA*	*NA*
Non-AJ Asian	<5/850	12/8203 (0.1%)	0.80 (0.10 – 6.19)	1
Non-AJ Chinese	<5/156	<5/1291	*NA*	*NA*
Non-AJ Mixed	<5/375	9/2322 (0.4%)	*NA*	*NA*
	Breast cancer			
AJ	11/131 (8.4%)	182/2595 (7.0%)	1.22 (0.64 – 2.29)	0.49
Non-AJ White	14/18683 (0.07%)	322/418255 (0.08%)	0.97 (0.57 – 1.66)	1
Non-AJ Black	<5/167	<5/7076	*NA*	*NA*
Non-AJ Asian	<5/232	13/8821 (0.1%)	*NA*	*NA*
Non-AJ Chinese	<5/48	<5/1399	*NA*	*NA*
Non-AJ Mixed	<5/102	9/2595 (0.4%)	*NA*	*NA*
	Prostate cancer			
AJ	7/85 (8.2%)	186/2641 (7.0%)	1.18 (0.54 – 2.60)	0.67
Non-AJ White	7/14159 (0.05%)	329/422779 (0.08%)	0.64 (0.30 – 1.34)	0.28
Non-AJ Black	<5/312	<5/6931	*NA*	*NA*
Non-AJ Asian	<5/137	12/8916 (0.1%)	5.46 (0.70 – 42.3)	0.18
Non-AJ Chinese	<5/11	<5/1436	*NA*	*NA*
Non-AJ Mixed	<5/46	9/2651 (0.3%)	*NA*	*NA*
	Pancreatic cancer			
AJ	<5/9	192/2717 (7.1%)	1.64 (0.20 – 13.21)	0.48
Non-AJ White	<5/1460	336/435478 (0.08%)	*NA*	*NA*
Non-AJ Black	<5/19	<5/7224	*NA*	*NA*
Non-AJ Asian	<5/15	13/9038 (0.1%)	*NA*	*NA*
Non-AJ Chinese	<5/<5	<5/1444	*NA*	*NA*
Non-AJ Mixed	<5/8	9/2689 (0.3%)	*NA*	*NA*

Supplementary Table 3: *APC* I1307K frequency in breast, prostate, pancreatic, and all cancer patients and patients without these phenotypes in the UK Biobank. Associated ORs for AJ and non-AJ white individuals, stratified by sex, are provided. P-values were obtained using a Fisher's exact test (Bonferroni-adjusted threshold of significance: $2.50x10^{-3}$).

**Ancestry**	**Sex**	**Cancer patients (I1307K/total)**	**Individuals w/o the corresponding cancer type (I1307K/total)**	**OR (95% CI)**	**Fisher's exact p-value**
		Colorectal cancer			
AJ	Female	<5/17	95/1474 (6.4%)	1.83 (0.42-8.02)	0.33
	Male	<5/22	96/1213 (7.9%)	*NA*	*NA*
Non-AJ White	Female	<5/3777	184/233233 (0.08%)	1.34 (0.50-3.62)	0.55
	Male	<5/4911	145/195017 (0.07%)	0.82 (0.26-2.58)	1
		All cancers			
AJ	Female	26/303 (8.6%)	67/1092 (6.1%)	1.39 (0.87-2.24)	0.16
	Male	19/235 (8.1%)	72/904 (8.0%)	1.02 (0.60-1.72)	1
Non-AJ White	Female	36/48846 (0.07%)	152/188164 (0.08%)	0.91 (0.63-1.30)	0.72
	Male	26/38843 (0.07%)	122/161085 (0.08%)	0.88 (0.58-1.35)	0.68
		Breast cancer			
AJ	Female	11/130 (8.5%)	86/1361 (6.3%)	1.34 (0.70-2.57)	0.36
	Male	<5/<5	89/1123 (7.9%)	*NA*	*NA*
Non-AJ White	Female	14/18577 (0.08%)	174/218433 (0.08%)	0.95 (0.55-1.63)	1
	Male	<5/106	148/199822 (0.07%)	*NA*	*NA*
		Prostate cancer			
AJ	Female	<5/<5	97/1490 (6.5%)	*NA*	*NA*
	Male	7/84 (8.3%)	89/1151 (7.7%)	1.08 (0.48-2.40)	0.83
Non-AJ White	Female	<5/<5	188/237010 (0.08%)	*NA*	*NA*
	Male	7/14159 (0.05%)	141/185769 (0.08%)	0.65 (0.30-1.39)	0.34
		Pancreatic cancer			
AJ	Female	<5/7	97/1484 (6.5%)	*NA*	*NA*
	Male	<5/<5	95/1233 (7.7%)	6.49 (0.58-72.22)	0.20
Non-AJ White	Female	<5/670	188/236340 (0.08%)	*NA*	*NA*
	Male	<5/790	148/199138 (0.07%)	*NA*	*NA*

Supplementary Table 4: *APC* I1307K frequency in breast, prostate, pancreatic, and all cancer patients and patients without these phenotypes in the UK Biobank. Associated ORs for AJ and non-AJ white individuals, stratified by age band, are provided. P-values were obtained using a Fisher's exact test (Bonferroni-adjusted threshold of significance: $1.00x10^{-3}$).

Ancestry	Age band	Cancer patients (I1307K/total)	Individuals w/o the corresponding cancer type (I1307K/total)	OR (95% CI)	Fisher's exact p-value
		Colorectal cancer			
AJ	**<50**	<5/<5	<5/<5	*NA*	*NA*
Non-AJ White	**<50**	<5/706	<5/602	*NA*	*NA*
AJ	**50-59**	<5/7	11/177 (6.2%)	4.60 (0.85-24.80)	0.11
Non-AJ White	**50-59**	<5/1938	52/56063 (0.09%)	1.11 (0.27-4.57)	0.70
AJ	**60-69**	<5/16	69/771 (8.9%)	*NA*	*NA*
Non-AJ White	**60-69**	<5/3427	101/130050 (0.08%)	1.13 (0.36-3.56)	0.75
AJ	**70-79**	<5/12	93/1451 (6.4%)	*NA*	*NA*
Non-AJ White	**70-79**	<5/2493	132/190552 (0.07%)	1.16 (0.29-4.68)	0.69
AJ	**≥80**	<5/<5	17/284 (6.0%)	*NA*	*NA*
Non-AJ White	**≥80**	<5/124	41/50983 (0.08%)	*NA*	*NA*
		All cancers			
AJ	**<50**	15/106 (14.2%)	<5/<5	*NA*	*NA*
Non-AJ White	**<50**	12/19462 (0.06%)	<5/413	0.13 (0.03-0.57)	0.03
AJ	**50-59**	8/117 (6.8%)	11/159 (6.9%)	0.99 (0.39-2.53)	1
Non-AJ White	**50-59**	17/20558 (0.08%)	47/50426 (0.09%)	0.89 (0.51-1.55)	0.78
AJ	**60-69**	15/199 (7.5%)	59/653 (9.0%)	0.83 (0.46-1.50)	0.67
Non-AJ White	**60-69**	19/29503 (0.06%)	91/109966 (0.08%)	0.78 (0.47-1.28)	0.35
AJ	**70-79**	6/113 (5.3%)	69/1154 (6.0%)	0.89 (0.38-2.09)	1
Non-AJ White	**70-79**	14/17554 (0.08%)	101/149453 (0.07%)	1.18 (0.67-2.06)	0.54
AJ	**≥80**	<5/<5	9/219 (4.1%)	8.11 (0.77-85.85)	0.16
Non-AJ White	**≥80**	<5/612	33/38991 (0.08%)	*NA*	*NA*
		Breast cancer			
AJ	**<50**	<5/29	<5/<5	*NA*	*NA*
Non-AJ White	**<50**	<5/4190	<5/567	0.05 (0.00-0.43)	0.006
AJ	**50-59**	<5/50	11/171 (6.4%)	1.24 (0.38-4.08)	0.75
Non-AJ White	**50-59**	6/6510 (0.09%)	52/55036 (0.09%)	0.98 (0.42-2.27)	1
AJ	**60-69**	<5/39	69/754 (9.2%)	0.84 (0.25-2.79)	1
Non-AJ White	**60-69**	<5/5768	101/127106 (0.08%)	0.87 (0.32-2.37)	1
AJ	**70-79**	<5/13	87/1389 (6.3%)	*NA*	*NA*
Non-AJ White	**70-79**	<5/2130	127/185751 (0.07%)	2.06 (0.66-6.48)	0.18
AJ	**≥80**	<5/<5	15/278 (5.4%)	*NA*	*NA*
Non-AJ White	**≥80**	<5/85	39/49795 (0.08%)	*NA*	*NA*
		Prostate cancer			
AJ	**<50**	<5/<5	<5/<5	*NA*	*NA*
Non-AJ White	**<50**	<5/83	<5/620	3.73 (0.34-41.64)	0.32
AJ	**50-59**	<5/6	11/179 (6.1%)	*NA*	*NA*
Non-AJ White	**50-59**	<5/2218	53/56320 (0.09%)	0.48 (0.07-3.47)	0.72
AJ	**60-69**	5/46 (10.9%)	70/773 (9.1%)	1.20 (0.46-3.12)	0.61
Non-AJ White	**60-69**	<5/7034	102/129852 (0.08%)	0.54 (0.17-1.71)	0.38

AJ	**70-79**	<5/32	89/1416 (6.3%)	0.99 (0.23-4.22)	1
Non-AJ White	**70-79**	<5/4718	131/186609 (0.07%)	0.60 (0.15-2.44)	0.78
AJ	**≥80**	<5/<5	15/269 (5.6%)	*NA*	*NA*
Non-AJ White	**≥80**	<5/106	41/49378 (0.08%)	*NA*	*NA*
		Pancreatic cancer			
AJ	**<50**	<5/<5	<5/<5	*NA*	*NA*
Non-AJ White	**<50**	<5/43	<5/609	*NA*	*NA*
AJ	**50-59**	<5/<5	11/179 (6.1%)	*NA*	*NA*
Non-AJ White	**50-59**	<5/170	53/56392 (0.09%)	*NA*	*NA*
AJ	**60-69**	<5/<5	71/776 (9.1%)	*NA*	*NA*
Non-AJ White	**60-69**	<5/565	103/131356 (0.08%)	*NA*	*NA*
AJ	**70-79**	<5/<5	92/1469 (6.3%)	3.99 (0.44-36.08)	0.26
Non-AJ White	**70-79**	<5/648	135/194523 (0.07%)	*NA*	*NA*
AJ	**≥80**	<5/<5	17/289 (5.9%)	*NA*	*NA*
Non-AJ White	**≥80**	<5/34	42/52598 (0.08%)	*NA*	*NA*

Supplementary Figure 1: Ethnicity breakdown of UK Biobank individuals included in analysis. Breakdown is by top-level ethnicity, and by PC-assessed AJ ancestry status.

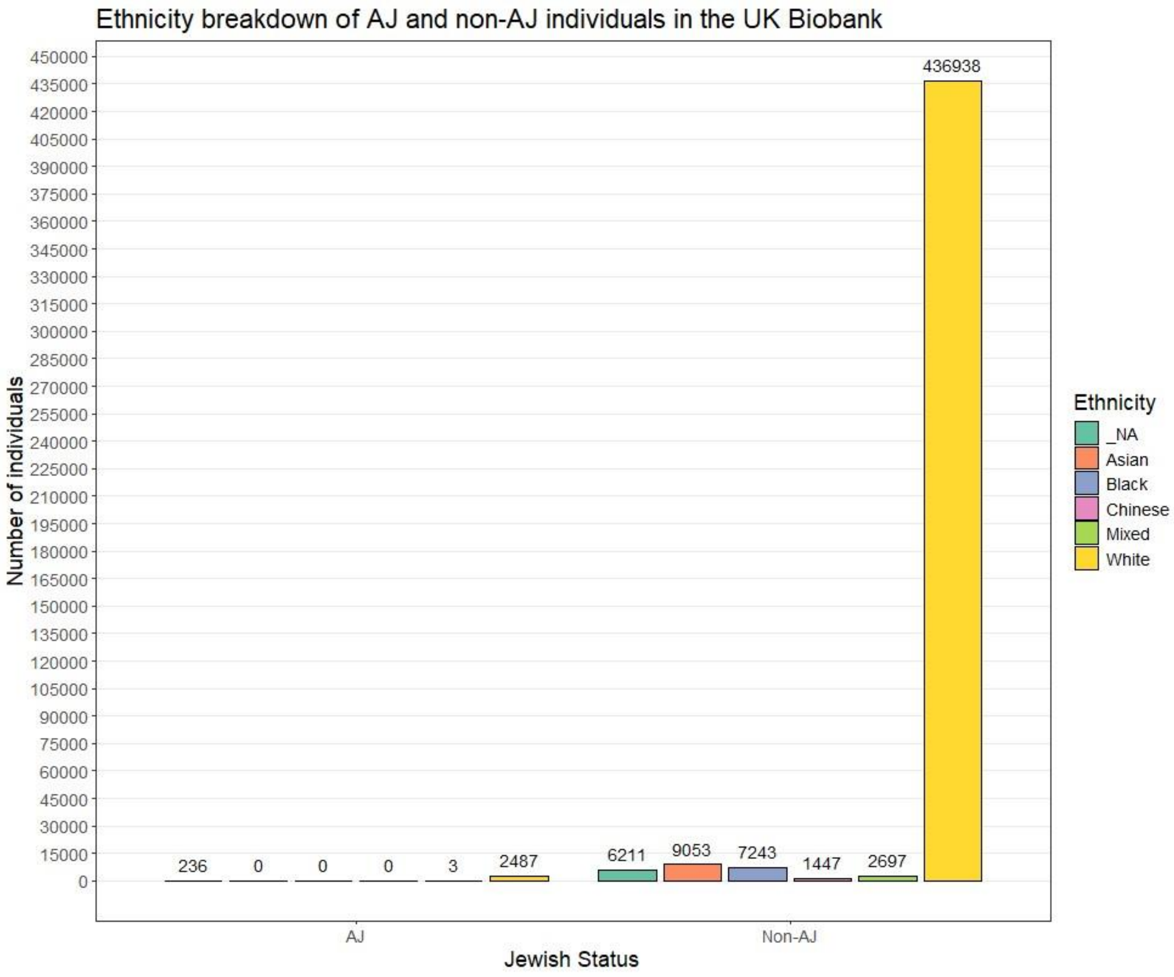

Supplement: online supplemental file 1 [file jmg-62-12-s001.pdf]
